# Comparison of two different host plant genera responding to grapevine leafroll-associated virus 3 infection

**DOI:** 10.1038/s41598-020-64972-8

**Published:** 2020-05-22

**Authors:** Cecilia A. Prator, Kar Mun Chooi, Dan Jones, Marcus W. Davy, Robin M. MacDiarmid, Rodrigo P. P. Almeida

**Affiliations:** 1 Department of Environmental Science, Policy, and Management, University of California, Berkeley, CA 94720 USA; 2 The New Zealand Institute for Plant and Food Research Limited, Auckland, New Zealand; 3 The New Zealand Institute for Plant and Food Research Limited, Te Puke, New Zealand

**Keywords:** Microbiology, Plant sciences

## Abstract

Grapevine leafroll-associated virus 3 (GLRaV-3) is one of the most important viruses of grapevine but, despite this, there remain several gaps in our understanding of its biology. Because of its narrow host range - limited to *Vitis* species - and because the virus is restricted to the phloem, most GLRaV-3 research has concentrated on epidemiology and the development of detection assays. The recent discovery that GLRaV-3 can infect *Nicotiana benthamiana*, a plant model organism, makes new opportunities available for research in this field. We used RNA-seq to compare both *V. vinifera* and P1/HC-Pro *N. benthamiana* host responses to GLRaV-3 infection. Our analysis revealed that the majority of DEGs observed between the two hosts were unique although responses between the two hosts also showed several shared gene expression results. When comparing gene expression patterns that were shared between the two hosts, we observed the downregulation of genes associated with stress chaperones, and the induction of gene families involved in primary plant physiological processes. This is the first analysis of gene expression profiles beyond *Vitis* to mealybug-transmitted GLRaV-3 and demonstrates that *N. benthamiana* could serve as a useful tool for future studies of GLRaV-3-host interactions.

## Introduction

One of the gaps in our knowledge of plant virology remains our understanding of how virus infection affects whole plants physiologically and biochemically, especially at the cellular level. Viruses are biotrophs, and plants respond with a highly polymorphic innate immune response to infection^1^. Determining how the plant responds to virus infection and which sets of genes are differentially expressed represents a first step to better understanding the mechanisms behind the regulatory pathways involved^2^. High throughput sequencing (HTS) based research has been increasingly carried out with model as well as in non-model plants for gene expression studies to aid in understanding host and virus responses during infection cycles^3^. Model organisms are indispensable to scientific progress because they are well studied and provide a biological setting to undertake experiments when ethics, costs, and technical difficulties can otherwise impair research. In the context of understanding virus infection in plants and in determining the usefulness of a model organism for future work, a critical comparison is of host response to virus infection between its original non-model host and a novel potential model organism.

Grapevine leafroll-associated virus 3 (GLRaV-3) was previously thought to be limited to *Vitis* species, but was recently demonstrated also to infect *Nicotiana benthamiana*^4^. GLRaV-3, a ssRNA virus within the family *Closteroviridae*, is regarded as the most important agent of grapevine leafroll disease (GLD) that results in substantial economic losses (20–40%) to the wine, table grape, raisin, and nursery industries^5^. The virus is transmitted primarily by phloem-sap sucking mealybugs (Hemiptera, Pseudococcidae) in a semi-persistent manner ^6^. It infects the phloem tissues of both *V. vinifera* and *N. benthamiana* hosts^4,6^. The finding that GLRaV-3 infects *N. benthamiana* provides advantages of working with this model organism when comparing time from infection to detection, relative ease of virion purification, as well as visualization of viral particles and virion structural proteins. Importantly, for *N. benthamiana* to be used as a model host for GLRaV-3 research it must share key responses with the natural grapevine host^4,7^.

Plant viruses cause significant changes in host gene expression in response to infection^8^. In recent years, RNA-sequencing technology has progressed rapidly, providing a more sensitive method to detect low-abundance host gene expression changes due to stresses induced by viral infection than previously observed using microarray technologies^9,10^. RNA-seq has quickly become the preferred tool for gene expression analyses in important model hosts like *N. benthamiana*. This popular experimental host has become an indispensable tool in plant virology because of its susceptibility to infection by a large number of diverse plant viruses, perhaps because of a naturally occurring mutation in an RNA-dependent RNA polymerase gene^7^. This, combined with the recent release of the draft genome sequence for *N. benthamiana*, has made it a particularly useful for host–pathogen studies focused on innate immunity and defense signaling, protein localization and interactions, and a system for protein expression and purification research^11,12^.

In contrast, gene expression studies in *V. vinifera* in response to viral infection are relatively limited. Most RNA-sequencing work on *V. vinifera* as a host has focused on differential gene expression analysis during berry development and other developmental stages of plant growth^3,13^. HTS has also been adapted for detection of virus infection in *V. vinifera*^14,15^. Transcriptome analyses and differential expression profiles of small RNAs associated with GLRaV-3 infection in grapes was recently found^16^. It is our hope that together with these studies, the gene expression profiles in response to GLRaV-3 infection in two distinct hosts will help gain a better understanding of host-pathogen interactions of GLD.

We used RNA-seq to analyze the gene expression profiles of *N. benthamiana* and *V. vinifera* responses to GLRaV-3 infection. This is a first assessment of how a novel host, other than *V. vinifera*, responds to GLRaV-3 infection. Responses between the two hosts show several shared gene expression results. This, together with the small number of shared gene expression differences, demonstrates that *N. benthamiana* could serve as a useful tool for future studies of GLRaV-3-host interactions.

## Results

RNAseq data were mapped to the respective *N. benthamiana* or *V. vinifera* genome and the results of that mapping assessed (Table 1). In all cases, the majority of sequenced reads (54–78%) mapped to the appropriate genome, although a proportion of reads mapped to multiple loci probably because of repeats or gene families. A substantial proportion of reads (22–46%) did not map to the genome. To determine whether RNA from other species had been included in the RNAseq data, analysis of ribosomal RNA was conducted, which demonstrated that cross-contamination between other organisms was not a major source of unmapped reads, and poor-quality sequence was removed as part of pre-processing.

**Table 1:** Mapping of RNA sequences to *Nicotiana benthamiana* and *Vitis vinifera*.

Species	Sample	Total reads	% mapped (uniquely)	% unmapped
*V. vinifera*	Healthy	17.5 Mbp	71 (60)	29
	Infected-1	43.3 Mbp	74 (67)	26
	Infected-2	6.5 Mbp	54 (46)	46
*N. benthamiana*	Healthy-1	52.7 Mbp	78 (66)	22
	Healthy-2	49.4 Mbp	76 (64)	24
	Infected	6.6 Mbp	63 (53)	37

Some sequencing libraries produced substantially less sequence than others, because of the difficulty of obtaining high quality RNA. Furthermore, not all treatments had replicates. However, differential gene expression analysis was performed using edgeR, with appropriate normalization, allowing for statistically valid gene expression comparisons. To determine if other viruses were present in our plant samples, we mapped reads from our sample sequences against a custom library of grapevine-infecting virus species and viroids (summarized in Supplementary Table S5). Interestingly, we observed a co-infection of GLRaV-3 with grapevine virus A in the infected *N. benthamiana* sample used in this study.

### *V. vinifera* response to GLRaV-3 infection

**Figure 1:**
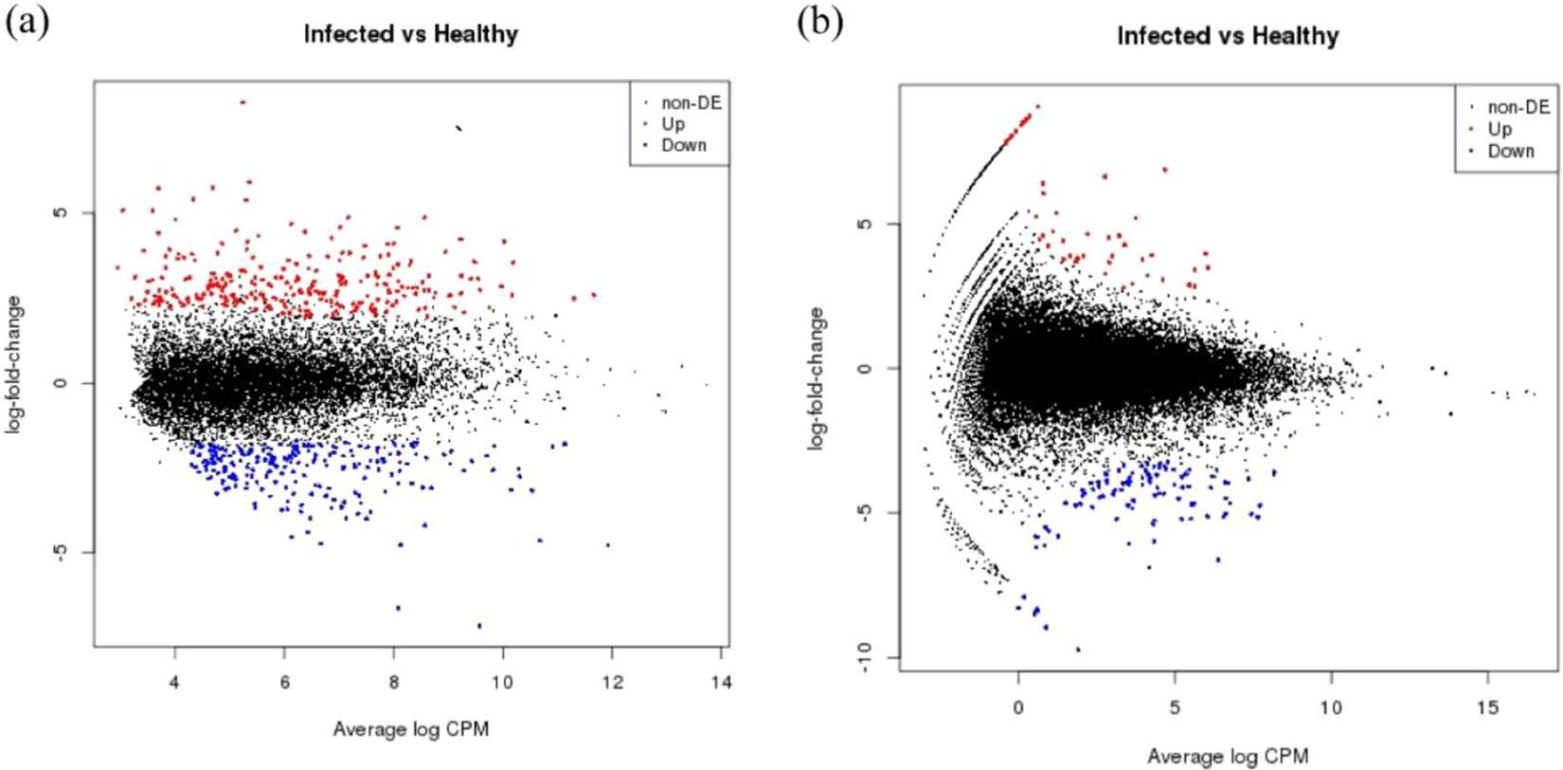
MA-plot of differential expression for (**a**) *Vitis vinifera* and (**b**) *Nicotiana benthamiana*, M-values, i.e. the log of the ratio of level counts for each gene between two samples against A-values. A-values, i.e. the average level counts for each gene across the two samples. (**a**) GLRaV-3 infected *V. vinifera* versus healthy *V. vinifera*. (**b**) GLRaV-3 infected N. benthamiana versus healthy *N. benthamiana*. Figure key: Non-DE, UP, and Down refer to Non-differentially expressed genes (black), Up regulated genes (red), and Down regulated genes (blue), respectively.

**Figure 2:**
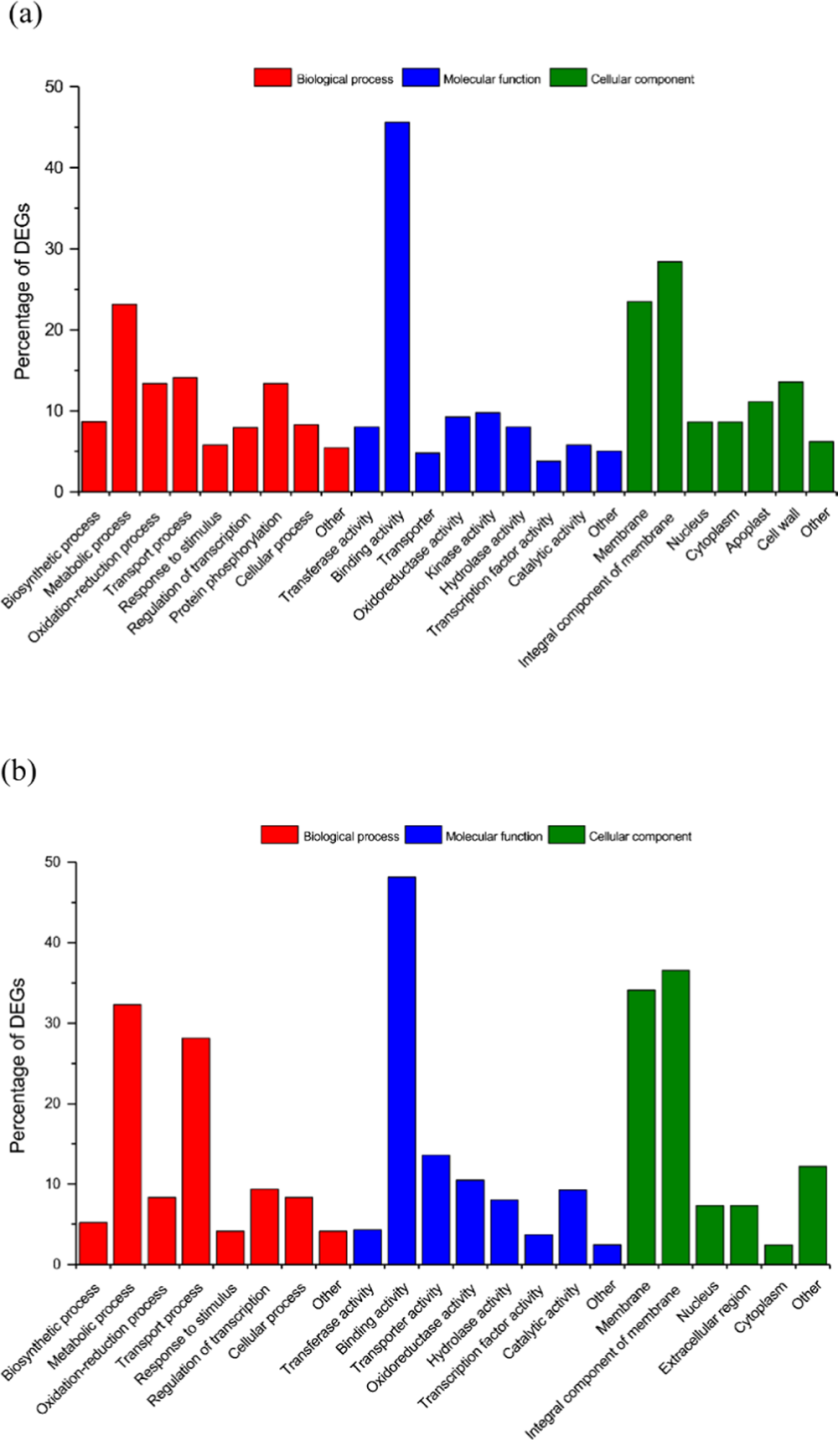
Functional categorization of up- and downregulated genes in (**a**) *Vitis vinifera* and (**b**) *Nicotiana benthamiana* in response to GLRaV-3 infection based on gene ontology annotations. For both *V. vinifera* and *N. benthamiana*, the differentially expressed genes (DEGs) were primarily related to “binding activity” molecular functions, mediate metabolic processes, and associated with the cellular membrane components.

In our analyses, 494 genes were differentially expressed in response to GRLaV-3 infection in *V. vinifera* (Fig. 1a, Supplementary Table S1). Of these differentially expressed genes (DEGs), 222 were downregulated and 272 were upregulated. Additionally, 44 genes were shown to be stably expressed when comparing infected versus healthy plants. Gene ontology (GO) analysis of the most commonly observed DEGs, revealed metabolic processes (biological process), binding activity (molecular function), and membrane and integral part of membrane (cellular component) to be the most common processes differentially expressed (Fig. 2a).

### *N. benthamiana* response to GLRaV-3 infection

In the herbaceous host, 157 genes were shown to be differentially expressed (Fig. 1b, Supplementary Table S1), of these, 107 were downregulated and 49 were upregulated, while 28 genes were stably expressed. In comparison to the trends observed in *V. vinifera* GO analysis, metabolic and transport processes were common biological processes observed, binding activity was a common molecular function, followed by membrane and integral part of membrane (cellular component) as the most common differentially expressed processes (Fig. 2b).

### Comparison of differential gene expression between *N. benthamiana* and *V. vinifera*

To determine if *N. benthamiana* could serve as a suitable model organism for this host-pathogen system, we determined how similarly it responded to GLRaV-3 infection compared with *V. vinifera*. General families of DEGs common in both hosts also followed similar expression changes, with six upregulated, seven downregulated, and two stably expressed genes in common (summarized in Table 2 with specific gene ID, transcript accumulation summarized in Supplementary Table S2). Only 1% of the shared DEGs observed in both hosts showed up- or downregulation patterns that were different from each other (Table 3). Shared genes were categorized into general groups and annotated with functions associated with expression based on literature searches. Several DEGs were shared but present in up (n = 10), down (n = 13), and/or stable (n = 3) categories concurrently (Supplementary Table S3).

**Table 2:** Summary of differentially expressed genes in common between *Nicotiana benthamiana* and *Vitis vinifera*.

Gene Name	Gene Function	Reference
***Upregulated***		
NAD(P)H-quinone oxidoreductase	Protect organisms from oxidative stress	Heyno *et al*.^49^
Subtilisin-like serine protease	Immune response	Figueiredo *et al*.^50^
Sugar transporter protein	Sugar transport, drought response	Yıldırım *et al*.^33^
U-box domain-containing protein	Degradation of aberrant proteins induced by stress	Azevedo *et al*.^51^
Beta-glucosidase	Initiate cell division	Brzobohaty *et al*.^52^
MLP-like protein	Drought tolerance	Wang *et al*.^53^
***Downregulated***		
BAG family molecular chaperone regulator	Regulate apoptosis-like processes	Doukhanina *et al*.^54^
Chaperone protein	Thermotolerance, protein disaggregation	Lee *et al*.^55^
Copper chaperone	Intracellular delivery of copper to target proteins	Harrison *et al*.^56^
Ethylene-responsive transcription factor	Transcription regulation	Fujimoto *et al*.^57^
Heat shock cognate 70 kDa	molecular chaperone, non-covalent folding/unfolding	Chen *et al*.^58^
Homeobox-leucine zipper protein	Drought tolerance	Lee & Chun^59^
Peptidyl-prolyl cis-trans isomerase	Thermotolerance, pH stabilization, biotic stress	Pogorelko *et al*.^60^
***Stable***		
60 S ribosomal protein	Defense against viral infection	Carvalho *et al*.^61^
Eukaryotic translation initiation factor	Abiotic stress tolerance	Gallino *et al*.^62^

**Table 3:** Summary of shared differentially expressed genes with different expression levels between *Nicotiana benthamiana and Vitis vinifera*.

Transcript ID	Gene Name	log2 FC	Gene Description	Reference
GSVIVG01007719001	Glutaredoxin	2.925504	Regulate cellular processes through dithiol–disulfide exchanges with many target proteins	Rouhier *et al*.^21^
GSVIVG01020614001	Glutaredoxin-C9	2.931628		
Niben101Scf06504g01026	Glutaredoxin family protein	−4.23343		
GSVIVG01019824001	RHOMBOID-like protein 2	3.539202	Catalyzes intramembrane proteolysis. May function in pollen elongation, signaling, development, apoptosis, and mitochondrial integrity	Knopf & Adam^22^
Niben101Scf18513g00008	RHOMBOID-like protein 14	−3.99537		
Niben101Scf03770g02001	RHOMBOID-like protein 14	−3.39187		
GSVIVG01009928001	Thaumatin-like protein 1b	−2.069703	Involved in anti-fungal response	Vigers *et al*.^20^
Niben101Scf01400g00014	Thaumatin-like protein	3.664741		
GSVIVG01026054001	UDP-glycosyltransferase 88F3	5.085967	Biosyntheses of cell-wall polysaccharides, the addition of N-linked glycans to glycoproteins, attachment of sugar moieties to various small molecules	Keegstra & Raikhel^23^
Niben101Scf02751g02006	UDP-Glycosyltransferase superfamily	−8.9506		
Niben101Scf04875g02008	UDP-Glycosyltransferase superfamily	−5.80191		
Niben101Scf06112g01008	UDP-Glycosyltransferase superfamily	−4.73772		

### RT-qPCR validation of selected genes

Validation of differentially expressed genes was performed by RT-qPCR. In total, eleven DEGs (seven grapevine and four *N. benthamiana*) were verified. Based on the transcriptome data, selection of genes whose expression did not vary between healthy and GLRaV-3-infected plants were used as references in RT-qPCR. All gene expression levels were consistent with the results of the transcriptome analysis for both hosts (Supplementary Fig. S1). Four of the grapevine genes (V8856.2, V8031, V6014, V9000) were consistently downregulated, while three grapevine genes (V2636, V1578, V9187) showed upregulation consistent with the transcriptome results. Of the four *N. benthamiana* genes verified, two genes (B1018, B2016) showed downregulation while the other two genes (B0002, B0006) showed upregulation consistent with the transcriptome analysis.

## Discussion

The discovery that GLRaV-3 could infect the model host *N. benthamiana* is an important finding for a research field where studies have been limited by a difficult host-pathogen system^4^. In this work, we compared the host responses between GLRaV-3-infected *V. vinifera* and P1/HC-Pro *N. benthamiana* to determine if gene expression profiles were similar. These are also the first gene expression data for GLRaV-3 infection in a host outside of *V. vinifera*.

Reads that did not map to either the *V. vinifera* or *N. benthamiana* genomes could be a result of the presence of RNA from other sources, e.g. RNA from GLRaV-3, other viruses, bacteria or fungi, or may be a consequence of poor quality sequence, or sequences that are correct but not present in the genome. The rRNA analysis to detect contamination from other organisms showed this as only a small contributor to unmatched reads. Therefore, it is likely that most unmapped reads do not map to the respective genome because that part of the genome is missing or is significantly different.

Data showed transcriptional changes in both GLRaV-3-infected *vs* healthy *V. vinifera* and infected *vs* healthy *N. benthamiana*. We observed 494 DEGs in infected *V. vinifera* compared with healthy plants. In contrast, only 157 DEGs were observed in GLRaV-3-infected *N. benthamiana*. Several variables could explain the increase in the number of DEGs expressed in *V. vinifera* compared with *N. benthamiana*. One limitation is the lack of a well-annotated genome for *N. benthamiana* relative to the more extensively annotated *V. vinifera* genome. As future research continues to improve available annotations for the *N. benthamiana* genome, more DEGs could be uncovered that were missed in our analysis. In other gene expression studies on *N. benthamiana* response to virus infection, smaller numbers of DEGs have been associated with infection of virus-resistant plant varieties as well as infected hosts with less severe observable symptoms^17–19^. In GLRaV-3-infected *N. benthamiana*, no clear symptoms were observed that could be attributed to GLRaV-3 infection when petioles were collected for analysis at two months post-inoculation. The smaller number of DEGs in *N. benthamiana* could be attributed to a lack of severe plant responses, as has been previously described in other virus infections in this host^17–19^. In addition, our results provide a limited snapshot of plant host response to GLRaV-3 infection taken during one timepoint. Future studies looking at multiple timepoints of virus infection as well as an increase in replicates used for sequencing could help to decipher this trend.

To determine if *N. benthamiana* could serve as a suitable model host for future studies, we wanted to determine how similar it responded to GLRaV-3 infection compared with *V. vinifera*. This also meant determining if there were any major differences in expression profiles between the two hosts. The majority of DEGs observed between the two hosts were unique. It should be noted that the comparison between hosts was limited by replication number available for sequencing. Additionally, P1/HC-Pro *N. benthamiana* was used in these studies because it was relatively easier to infect with GLRaV-3 than wild-type tobacco^4^. It is likely that the expression of a silencing suppressor interferes with how GLRaV-3 establishes infection and our results reflect the host response from the transgenic host. The infected *N. benthamiana* was co-infected with GLRaV-3 with grapevine virus A. It is possible that the co-infection also impacted host gene regulation. Among the shared DEGs between the two hosts, only 1% in *N. benthamiana* showed up- or downregulation that was different from those observed in *V. vinifera*. Only one of these, thaumatin-like protein, which was downregulated in *V. vinifera* and upregulated in *N. benthamiana*, is found to be associated with response to biotic stress and in this case is thought to be antifungal^20^. The other three gene families detected - glutaredoxin, RHOMBOID-like protein, and UDP-glycosyltransferase - are involved in other plant cellular processes^21–23^. This small number of genes observed to have differing gene expression, and the fact that only one appears to be related to biotic stress, is encouraging for future work to determine the use of *N. benthamiana* as a model host for this system.

Response to GLRaV-3 infection resulted in a common set of genes being differentially expressed in both hosts. When comparing gene expression patterns that were shared between the two hosts, two outcomes were observed: (i) the downregulation of genes associated with stress chaperones, and (ii) the induction of gene families involved in primary plant physiological processes. We observed a shared pattern of downregulated genes associated with drought stress. Among these downregulated genes were molecular chaperones and specifically HSP70, which is part of a larger group of heat shock proteins. Chaperone synthesis is a common aspect of plant virus infection, and the induction of biotic and abiotic stress response genes can be associated with exposure to other various stressors, including thermal changes, heavy metal accumulation, pH variation, and hypoxia^19,24–26^. Our samples used for sequencing were collected at two months post-inoculation when virus infection would have been well established throughout the plant. It is possible that downregulation of genes associated with a stress response could be the result of a successful long-term GLRaV-3 infection, as a strategy to escape plant defenses. The downregulation of stress-related genes has also been described in prunus necrotic ringspot ilarvirus infection of *N. benthamiana*, where mild symptoms were observed in comparison with responses to other viruses infecting this host^19^. The lack of observable symptoms associated with GLRaV-3 infection at two months post-inoculation in both hosts could perhaps be attributed to the escape of plant stress responses.

The shared downregulation of HSP70s observed in both hosts is another example of a molecular chaperone commonly associated with host response to stress and viral infection being repressed. Recently, it has been shown that heat shock proteins are associated with regulation of the plant immune response, and HSP70 as well as other chaperones have been shown to be transiently up- or downregulated in a dynamic manner through different stages of viral infection^27–29^. Many plant and animal viruses recruit host cellular HSP70s for replication, and cytoplasmic HSP70 has also been shown to enhance infection of *N. benthamiana* by tobacco mosaic virus, potato virus X, and watermelon mosaic virus^29^. Although HSP70s appear to be an important factor for plant-virus infections, viruses do not typically encode their own HSP70s and must rely on their availability in an infected host^30^. In contrast, viruses from the family *Closteroviridae* encode their own homologs of HSP70s (HSP70h)^30^. For GLRaV-3, HSP70h is thought to be associated with cell-to-cell movement and virion assembly^31^. Because GLRaV-3 encodes its own customized HSP70h, it does not have to rely on the host to provide this protein, thus explaining a possible model for the downregulation of these genes in *N. benthamiana* and *V. vinifera*. This could also be another strategy used by the virus to avoid host stress responses.

Both *V. vinifera* and *N. benthamiana* share upregulation of a few genes associated with general plant or cellular processes, such as sugar transport and ubiquitination proteins. The induction of U-box domain-containing proteins in both hosts could be interpreted as a mechanism to cope with GLRaV-3 infection. The initiation of plant immune responses requires ubiquitination for positive and negative regulation, and is also involved in hormone signaling required for cellular integration of biotic stress cues^32^. Recent studies have also demonstrated that ubiquitination-associated proteins are targeted by pathogen virulence effectors, emphasizing their importance in immunity^32^.

Another commonly upregulated family of proteins was associated with sugar transport. This group of proteins is associated with drought stress as well as cellular sugar transport^33^. Accumulation of soluble sugars, decreased photosynthesis and increased respiration have been linked to virus infection in plants^34^. Previous work has also demonstrated that GLRaV-3 infection induces genes related to sugar metabolism, such as sugar transporters and glycosyl transferases^35^. In GLRaV-3 infection of grapevines, it is common to observe symptoms like leaf curling that are associated with decreased photosynthesis^36^. This is thought to be due to the movement and accumulation of sugar to the roots of the plant. Since GLRaV-3 affects sugar accumulation^37^, it is therefore not surprising to see an upregulation of sugar transport proteins in hosts infected by GLRaV-3. The upregulation of genes involved in this pathway in *N. benthamiana* demonstrates that the virus also has an effect on sugar transport in the herbaceous host. Future experiments should measure the sugar content of roots in GLRaV-3-infected *N. benthamiana*.

*N. benthamiana* has already shown several promising advantages over *V. vinifera* to serve as a model organism, including a shortened time from infection to detection, relative ease of virion purifications, as well as visualization of viral particles and structural proteins^4^. Model systems are essential to the propagation of knowledge when ethics, costs, and technical difficulties can be impediments to experiments^38,39^. In addition, *N. benthamiana* is an herbaceous plant, capable of being grown in greenhouse conditions year around, compared with *V. vinifera*, a deciduous, woody host, where transmission experiments are limited by growing season. Ease of genetic transformations methods and use for virus-induced gene silencing or transient protein expression, make *N. benthamiana* a popular choice as a tool in plant biology^7^. Further experiments are needed to increase efficiencies in the initial virus transmission experiments to wild-type and/or transgenic *N. benthamiana* to allow for comparisons with non-transgenic plants and with larger sample sizes.

In conclusion, our results indicate that *N. benthamiana* and *V. vinifera* show a promising degree of similar gene expression patterns in response to GLRaV-3 infection, although many of the DEGs observed were unique to each respective host. As research on genome annotation of both organisms progresses, we expect further interesting insights into host response to GLRaV-3 infection in the future. To help to continue our understanding of this disease, future work could isolate proteins of interest for reverse genetic experiments to test for roles in viral pathogenesis and to provide insights into signaling pathways that are affected by GLRaV-3 infection.

## Materials and Methods

### Plants used for the analysis

*Planococcus ficus* (Hemiptera, Pseudococcidae) colonies were maintained on butternut squash (*Cucurbita moschata*) at 22 °C, with a 16:8-h photoperiod. First instar mealybugs were used for all experiments because they were shown to be the most efficient life stages for transmitting GLRaV-3^6^. Wet Whatman filter papers were placed on top of mealybug colonies. After 30 min the papers were pinned to GLRaV-3 source vine cuttings (accession LR101 (cv. Italia-3), group I) provided by Foundation Plant Services, University of California Davis, CA. After a 24-h acquisition access period (AAP), approximately 20 first instars were transferred manually with a small paintbrush to either healthy *V. vinifera* (cv. Cabernet Sauvignon) or to *N. benthamiana* expressing the turnip mosaic virus P1/HC-Pro, kindly supplied by B. Falk (University of California, Davis). Transgenic tobacco were used because transmission efficiencies to wildtype tobacco were very low and previously published studies showed increased numbers of P1/HC-Pro *N. benthamiana* plants infected (n = 11 total over the course of experiments) when compared to wildtype *N. benthamiana* (n = 1)^4^. After 4 days, any visible mealybugs were removed and plants were sprayed with insecticide before being moved to the greenhouse. To test for GLRaV-3 infection, petiole samples were collected from plants at two months post-inoculation and RNA extractions were completed on 100 mg of petiole tissue^40^. One-step reverse transcription-polymerase chain reaction (RT-PCR) was then performed and PCR products were analyzed using fragment analysis as described previously^40^. At two months post inoculation for this experiment, petioles from four non-infected *V. vinifera*, four GLRaV-3-infected *V. vinifera*, three non-infected *N. benthamiana*, and four GLRaV-3-infected *N. benthamiana* were collected for RNA extractions and HTS submission.

### RNA extractions

0.1 g of petioles from a known GLRaV-3-infected *N. benthamiana* plant and 0.1 g petioles from the original GLRaV-3 source *V. vinifera* were used for next generation sequencing. For RNA extractions, petioles were ground in liquid nitrogen and added to 5 mL of Guanidine extraction buffer (4 M Guanidine thiocyanate, 0.2 M sodium acetate, 25 mM EDTA, 2.5% polyvinylpyrrolidone-40) and 1% beta-mercaptoethanol. 20% sarcosyl buffer was added followed by vigorous mixing and incubation in a 57 °C water bath for 12 minutes, vortexing every 3 minutes for better lysis efficiency. The extract was then added to QIAshredder columns (Qiagen) and the remainder of the protocol was followed according to Qiagen RNeasy Plant Mini Kit instructions as previously described^41^. RNA concentration and quality were evaluated by measuring the absorbance at 260 nm and the absorbance ratio 260/280 with a NanoDrop 2000 spectrophotometer (Thermo Fisher Scientific, USA) and stored at −80 °C until further analysis.

### RNASeq library preparation and sequencing

Sequencing libraries were constructed at the Functional Genomics Lab (FGL), a QB3-Berkeley Core Research Facility (University of California, Berkeley). Quality of RNA was checked on a 2100 Bioanalyzer (Agilent Technologies, CA, USA). The library preparation was done using RiboZero and Apollo 324 with PrepX RNAseq Library Prep Kits (WaferGen Biosystems, Fremont, CA) and 15 cycles of PCR amplification was used for index addition and library fragment enrichment. RNA samples with >RIN 8 were selected for sequencing. From this selection, one healthy *V. vinifera*, two GRLaV-3-infected *V. vinifera*, two healthy *N. benthamiana*, and one GLRaV-3-infected samples were sequenced.

Genomic sequencing (150 bp paired-end) was done using the Illumina platform (Illumina, Inc., CA, USA) at Vincent J. Coates Genomics Sequencing Laboratory at University of California, Berkeley. The raw data are publicly available as NCBI BioSample accession numbers: SAMN10753963, SAMN10753964, SAMN10753965, SAMN10753966, SAMN10753967, SAMN10753968. To determine if viruses other than GLRaV-3 were present in our samples, trimmed sequences (as described below) against a custom library of 57 grapevine virus species (included representatives of each GLRaV-3 genotype known to-date) and four viroids found in grapevine^42^. Briefly, trimmed reads were aligned to a grapevine or *N. benthamiana* genome and the unaligned reads were mapped against the virus and viroid library using Bowtie2 ver. 2.2.9^43^.

### Quality assessment and pre-processing

Sequence data were assessed for overall quality using FastQC (0.11.7) and MultiQC (1.5), before and after every pre-processing step. Ribosomal RNA was removed using SortMeRNA (2.1), and the samples checked for the presence of contaminant ribosomal RNA. Sequences were trimmed by quality and Illumina adaptors removed using Trimmomatic (0.36). This workflow (including MultiQC reports) is described in full in the github repository https://github.com/PlantandFoodResearch/bioinf_Vitis_Nicotiana_RNAseq (Refer to Jupyter notebook and MultiQC report files: 01_RNAseq_Quality_Control_Vitis_Nicotiana-Manuscript.ipynb).

### Reference-based gene expression

Gene expression in both hosts was assessed in reference to existing genomes and annotations. In *N. benthamiana*, the Niben 1.0.1 genome and annotations were obtained from solgenomics.net (https://solgenomics.net/organism/Nicotiana_benthamiana/genome)^44^. For *V. vinifera*, the Genoscope 12X genome and annotations were obtained from Genoscope (www.genoscope.cns.fr/externe/GenomeBrowser/Vitis/). For both species, pre-processed RNAseq reads were mapped to the appropriate genome using STAR (2.6.1a), deriving raw read counts for each annotated gene in each sample. These workflows are described in full (Refer to Jupyter notebook, Files 02a_RNAseq_Alignment_to_Reference_Nicotinia_benthamiana-Manuscript.ipynb, 02b_RNAseq_Alignment_to_Reference_Vitis_vinifera-Manuscript.ipynb).

### Differential gene expression

Differential gene expression between healthy and infected samples was assessed for both species using edgeR^45^. Briefly, read counts are normalized for library size using the trimmed mean of m-values^45^, genes that showed very low abundance were removed, and differential expression assessed using both the likelihood ratio and quasi-likelihood F-test methods. These workflows are described in full (Refer to R markdown notebooks, Files GRLaV_Nb_EdgeR.Rmd, GRLaV_Vv_EdgeR.Rmd).

### Quantitative RT-PCR

A total of 11 DEGs (seven *V. vinifera* and four *N. benthamiana* genes), and four stable expressed genes (two per plant host) identified by RNA-Seq were selected for validating differential expression analysis by RT-qPCR (Supplementary Table S4). Primers were designed using GenScript Real-time PCR (TaqMan) Primer Design online tool (https://www.genscript.com/tools/real-time-pcr-tagman-primer-design-tool) requiring one primer pair to cross an exon-exon junction. The OligoAnalyzer 3.1 program (Integrated DNA Technologies, Coralville, USA) was used to analyze the likelihood of prospective primers generating secondary structures.

Total RNAs from petioles from four non-infected *V. vinifera*, four GLRaV-3-infected *V. vinifera*, three non-infected *N. benthamiana*, and four GLRaV-3-infected *N. benthamiana* were collected for RNA extractions and isolated using a modified Qiagen kit protocol, described above. Amplicons were synthesized using two-step qualitative RT-PCR (RT-qPCR), with Superscript III Reverse Transcriptase (Invitrogen, Carlsbad, CA) used to synthesize the first-strand cDNA, following treatment with Invitrogen RNaseOUT Recombinant Ribonuclease Inhibitor (Invitrogen, Carlsbad, CA). qPCR was carried out using PerfeCTa SYBR Green SuperMix (Quanta Biosciences, Inc., Gaithersburg, MD) on a LightCycler 480 System (Roche Diagnostics, Penzberg, Germany) under the following conditions: 95 °C for 5 min, 40 cycles of 95 °C for 10 s, 60 °C for 20 s and 72 °C for 20 s, followed by a dissociation step. The Ct values and amplification curve data for each reaction generated by the Lightcycler 480 software were exported. Relative quantification (RQ) values for each sample were calculated using the method described by Pfaffl, and the geometric average of the plant genes showing stable expression by HTS was used to normalize the data, instead of using a single reference gene^46–48^. Calculated RQ values were log2 transformed and averaged for each of the technical replications of each sample and primer pair combination.

## Supplementary information

Supplementary Information

Supplementary Table S1

Supplementary Table S2

Supplementary Table S5
