# Supplementary material for: Comparison of two different host plant genera responding to grapevine leafroll-associated virus 3 infection
Source: Sci Rep. 2020 May 22;10:8505. doi: 10.1038/s41598-020-64972-8 (PMC7244584; doi:10.1038/s41598-020-64972-8)
Supplement: Supplementary file 1 — Supplementary Information [file 41598_2020_64972_MOESM1_ESM.pdf]

Comparison of two different host plant genera responding to grapevine leafroll-associated virus 3 infection

Cecilia A. Prator[a], Kar Mun Chooi[b], Dan Jones[b], Marcus W. Davy[c], Robin MacDiarmid[b], Rodrigo P.P. Almeida[a]*

[a]Department of Environmental Science, Policy, and Management, University of California, Berkeley, CA 94720, USA

[b]The New Zealand Institute for Plant and Food Research Limited, Auckland, New Zealand

[c]The New Zealand Institute for Plant and Food Research Limited, Te Puke, New Zealand

#Address correspondence to Rodrigo P.P. Almeida, rodrigoalmeida@berkeley.edu

SUPPLEMENTARY FIGURE LEGENDS

Supplementary Figure S1. RT-qPCR validation of differentially expressed genes. Log2 fold expression values of (a) seven *Vitis vinifera* and (b) four *Nicotiana benthamiana* RNA-seq-selected genes.

Supplementary Table S1. Summary of differentially expressed genes

Supplementary Table S2. Summary of transcript accumulation

Supplementary Table S3. Summary of shared differentially expressed genes found in more than one expression category to be up, down, or stably expressed.

Supplementary Table S4. Real-time polymerase chain reaction primers and gene targets.

Supplementary Table S5. Summary of viruses and viroids present in plant samples.

Supplementary Figure S1

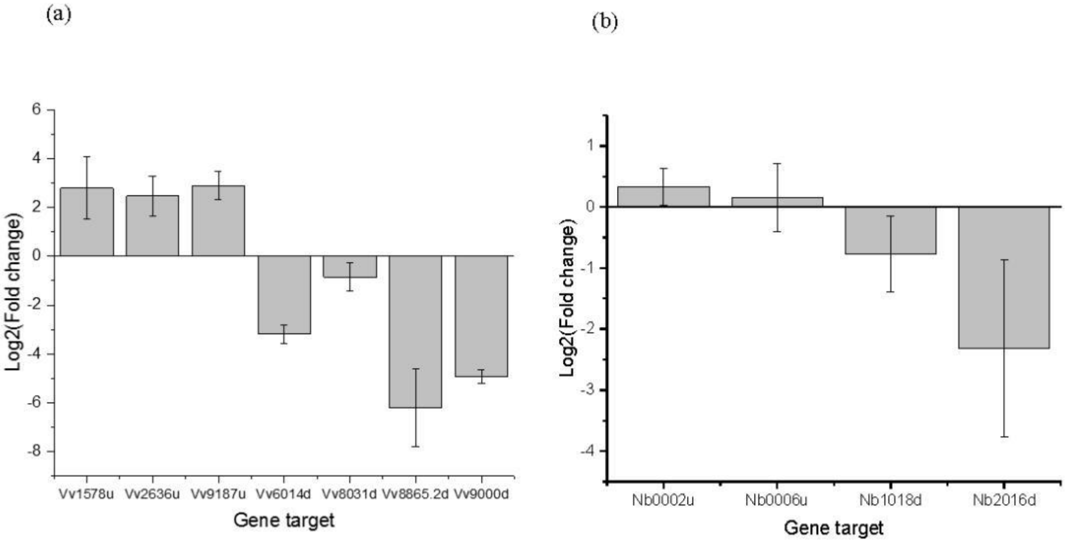

Table S3. Summary of shared differentially expressed genes found in more than one expression category to be up, down, or stably expressed.

**Upregulated**
Auxin transporter-like protein
Cytochrome P450
NAC domain-containing protein
NAD(P)H-quinone oxidoreductase
Protein NRT1/ PTR
Subtilisin-like serine protease
Sugar transporter protein
Sulfate transporter
U-box domain-containing protein
Zinc finger protein

**Downregulated**
17.4 kDa class III heat shock protein
Auxin efflux carrier component
BAG family molecular chaperone regulator
Chaperone protein
Copper chaperone
Cytochrome P450
Ethylene-responsive transcription factor
Heat shock cognate 70 kDa
Homeobox-leucine zipper protein
Peptidyl-prolyl cis-trans isomerase
Protein NRT1/ PTR
Sulfate transporter
Zinc finger protein

**Stable**
60S ribosomal protein
Eukaryotic translation initiation factor
NAC domain-containing protein

Table S4. Real-time polymerase chain reaction primers and gene targets.

Primer name	Nucleotide sequence (5'-3')	Transcript ID	Description
Vitis vinifera			
V8856.2F	AGCTGGTGGGTCATTCACTG	GSVIVG01028856001	heat shock protein 83
V8856.2R	GTACTCCACCTGGTCGTCCT		
V8031F	ACGTAGACAAGAAGCTCGCTGA	GSVIVG01038031001	probable glutathione S-transferase parC
V8031R	AGAGAAGGGCACCAGAGCCA		
V2636F	CAATGTGATGGAGGCGAAGG	GSVIVG01012636001	thiamine thiazole synthase 2, chloroplastic
V2636R	GGTAGGACCCATCCTTGGAG		
V1578F	AGCAGGGTATCCTCCACAAC	GSVIVG01015780001	cysteine-rich and transmembrane domain-containing protein A
V1578R	CAAGGCAGCACAACATCCTT		
V9187F	TGGGATTGTCAACAGGCTCT	GSVIVG01009187001	VIN3-like protein 2 isoform X1
V9187R	TCGTATCTGCATCCCTGCTT		
V6014F	TGCGGTTCAGGCTGGTGTTT	GSVIVG01026014001	stromal 70 kDa heat shock-related protein, chloroplastic
V6014R	TTTGCCCATCTGCTGCCGTA		
V9000F	ACAACCCAAGGTGGTAGGTG	GSVIVG01035900001	chaperone protein ClpB4, mitochondrial
V9000R	AATAGTTGCCGCCCAAACCT		
V3149F*	ACTGCGTGTTGGAGTAAGGA	GSVIVG01023149001	auxin response factor 1 isoform X1
V3149R*	CGACCGACTTGTTCTTGGTT		
V1341F*	TGGGAAGAGATCCAGGCAGCA	GSVIVG01013410001	Dolichyl-diphosphooligosaccharide--protein glycosyltransferase
V1341R*	GCTCCTCCACCTTGGGCAAT		
Nicotiana benthamiana			
B1018F	TTGCCTTTGGTGTCCTTGGT	Niben101Scf01182g01018	Bidirectional sugar transporter N3
B1018R	TGAGGAGTGCAGTGTTGGTC		
B2016F	TGCGAGTCGAACGAACTGCT	Niben101Scf00739g02016	Zinc finger Ran-binding domain-containing protein 2
B2016R	CATACCACATCCAATTCTGCTGC		
B0002F	ACCACCAGGATTTAGGTTCTTTCCT	Niben101Scf03366g00002	NAC domain-containing protein 21/2
B0002R	GCCACCTCAGGAAGTTGCCA		
B0006F	TGTCTCTCAGGCTCACCACGA	Niben101Scf03243g00006	Tubulin alpha chain
B0006R	TTCACGTCCTTGGGCACCAC		
B0009F*	GCTTGGAGAAGCGGTTTGGTC	Niben101Scf07824g00009	cellulose synthase 1
B0009R*	GCCCACCGAAGCACCTCTTT		
B0007F*	TGTCCAGGAGAGATTCGGGTCTG	Niben101Scf06579g00007	Cytochrome b-c1 complex subunit 7
B0007R*	ACACGCACGCTCTGTGTCTC		

* genes showing stable expression by HTS used to normalize data
